# KEGNI: knowledge graph enhanced framework for gene regulatory network inference

**DOI:** 10.1186/s13059-025-03780-7

**Published:** 2025-09-22

**Authors:** Pengxiao Li, Lin Li, Jingminjie Nan, Jiahuan Chen, Jielin Sun, Yanan Cao

**Affiliations:** 1 Shanghai Center for Systems Biomedicine, Key Laboratory of Systems Biomedicine (Ministry of Education), Institute of Translational Medicine, Shanghai Jiao Tong University, Shanghai, China; 2 Ruijin Yangtze River Delta Health Institute, Wuxi Branch of Ruijin Hospital, Ruijin Hospital, Shanghai Jiao Tong University School of Medicine, Shanghai, China; 3 Department of Endocrine and Metabolic Diseases, Shanghai Institute of Endocrine and Metabolic Diseases, Ruijin Hospital, Shanghai Jiao Tong University School of Medicine, Shanghai, China

**Keywords:** Gene regulatory networks, ScRNA-seq, Knowledge graph, Self-supervised learning, Multi-task learning

## Abstract

Inference of cell type-specific gene regulatory networks (GRNs) is a fundamental step in investigating complex regulatory mechanisms. Here, we present KEGNI (Knowledge graph-Enhanced Gene regulatory Network Inference), a knowledge-guided framework that employs a graph autoencoder to capture gene regulatory relationships and incorporates a knowledge graph to infer GRNs based on scRNA-seq data. KEGNI shows superior performance compared to multiple methods using scRNA-seq data or paired scRNA-seq and scATAC-seq data. KEGNI can identify driver genes and elucidate the regulatory mechanisms underlying distinct cellular contexts. The modular design of KEGNI supports the integration of various knowledge graphs for context-specific tasks.

**Supplementary Information:**

The online version contains supplementary material available at 10.1186/s13059-025-03780-7.

## Background

The gene regulatory networks (GRNs) encompass the complex interactions of genes and regulators in cells [1–4], which is essential for understanding the control and dynamics of cellular mechanisms in physiological and pathological processes [5–7]. Single-cell sequencing technologies have enabled cell type-specific GRNs inference and facilitated the development of network inference utilizing single-cell omics data. Numerous algorithms have been developed, such as PIDC [8], SCENIC [9], GENIE3 [10], and GRNBoost2 [11] based on gene co-expression patterns from single-cell RNA sequencing (scRNA-seq) data. However, this assumption may lead to an increase in false positives, as not all predicted correlations are causal relationships. Recent deep learning-based computational strategies have demonstrated strong capabilities in capturing complex and nonlinear dependencies from gene expression data. For instance, scGeneRAI employs an interpretable framework based on layer-wise relevance propagation to infer GRNs [12]. STGRNS is a transformer-based method that infers gene regulatory networks based on known relationships between genes [13]. GENELink [14], GNNLink [15], and AttentionGRN [16] utilize graph neural network architectures to integrate both topological and contextual information. CNNC [17], DeepDRIM [18], and DeepIMAGER [19] transform gene pairs into image-like representations and apply convolutional neural networks to capture higher-order gene interactions, overcoming limitations of traditional co-expression-based methods. Moreover, FigR [20], SCENIC + [21], LINGER [22], and scMultiomeGRN [23] incorporate external knowledge or ATAC-seq data to enhance the accuracy of GRN inference and effectively reduce false positives [24, 25].

However, obstacles still need to be resolved for the GRN inference. The epigenetic data are often insufficient for many cell types. The integration of unpaired scRNA-seq and scATAC-seq data by additional tools increases the risk of extra noise [26–28]. Moreover, the initial graph structure used for GRN inference is frequently built from prior gene interactions obtained from databases such as TRRUST [29, 30], RegNetwork [31], and KEGG [32, 33]. Consequently, the initial graph, which typically formulates the GRN as a link prediction task [14, 34], may fail to accurately capture cell type-specific regulatory interactions. Hence, the method of constructing comprehensive GRNs using scRNA-seq data and external information is needed to overcome these challenges. Here, we designed the computational framework KEGNI (Knowledge-graph Enhanced Gene Network Inference), an end-to-end framework for cell type-specific GRN inference based on scRNA-seq data and integration of reliable gene or protein interactions.

To enhance the performance of GRN construction, KEGNI employs a graph autoencoder to capture relationships between genes from expression profiles, in which genes are nodes and gene expressions are features. Given the gene expression data directly reflect the biological signals, KEGNI adopts a generative self-supervised learning strategy based on gene expression features. Inspired by the GraphMAE [35], KEGNI reconstructs the expression of randomly masked genes to effectively learn gene representations. A knowledge graph is constructed to integrate external knowledge into self-supervised graph autoencoder, and contrastive learning with negative sampling is used for knowledge graph embedding. We compared the performance of KEGNI with 8 methods using the BEELINE framework [36], which was designed to assess the accuracy, robustness, and efficiency of GRN inference techniques based on scRNA-seq benchmark datasets. Additionally, we compared KEGNI with 4 methods based on paired scRNA-seq and scATAC-seq data. Altogether, KEGNI demonstrates its superior performance on precise GRN construction for identification of key regulatory drivers and mechanisms under different conditions.

## Results

### The KEGNI framework

**Fig. 1:**
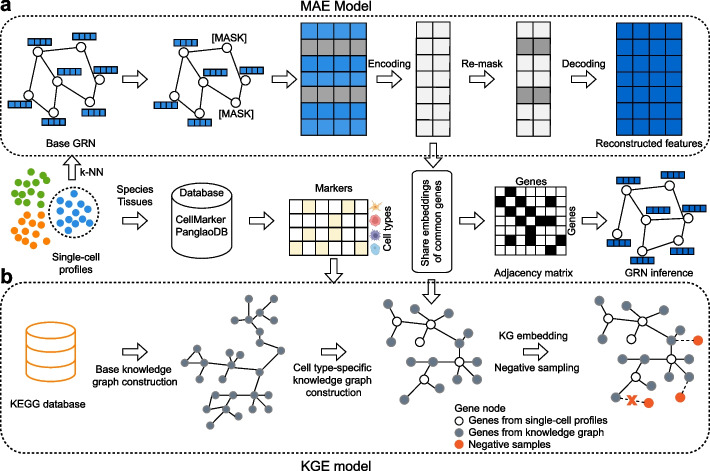
Schematic overview of the KEGNI framework. KEGNI is a comprehensive framework that integrates a Masked Autoencoder (MAE) model and a Knowledge Graph Embedding (KGE) model. **a** A base GRN is constructed from scRNA-seq data of a specific cell population using *k*-nearest neighbors (k-NN). The MAE takes this base GRN as input and focuses on the reconstruction of masked node features. **b** A cell type-specific knowledge graph is built using KEGG pathway information and relevant cell type markers. The KGE model employs a contrastive learning approach with negative sampling to achieve knowledge graph embedding. Nodes in the graph represent genes. Black hollow circles indicate genes from single-cell profiles; gray solid circles represent genes from the knowledge graph; red solid circles denote negative samples. Common genes are those shared between the single-cell expression profiles and the cell type-specific knowledge graph

The integrated framework of KEGNI is designed to infer cell type-specific GRNs by two model components, the Masked Graph Autoencoders (MAE) model for extracting gene relationships from scRNA-seq data (Fig. 1a) and the Knowledge Graph Embedding (KGE) model for leveraging prior biological knowledge (Fig. 1b). Initially, a base graph was constructed using the *k*-nearest neighbors (k-NN) algorithm based on Euclidean distances computed by gene expression profiles with cell type annotations. Each gene is represented as a node with expression levels as features.

Subsequently, the MAE model uses the graph as input for learning hidden gene representations through a self-supervised learning strategy, which randomly masks a subset of node features and takes their reconstruction as the objective. The KGE model uses a cell type-specific knowledge graph as input and employs a contrastive learning approach to enhance the performance of GRN inference with prior knowledge. The knowledge graph was constructed based on the KEGG PATHWAY database [32] and refined by selecting relevant nodes and edges with cell type markers, identified from the CellMarker 2.0 database [37] (see Methods; Additional file 1: Supplementary Notes 1 and 2). Finally, the KEGNI employs a multi-task learning approach that jointly optimizes the objectives of MAE and KGE models. For common genes in scRNA-seq data and cell type-specific knowledge graph, embeddings learned by the MAE model are shared with the KGE model and jointly updated under the objectives of both models. Additionally, KEGNI allows the MAE model to be used independently in the framework.

### KEGNI improves the cell type-specific GRN inference

To assess the performance of KEGNI in the GRN inference, we compared the KEGNI and MAE model with previous methods. We first employed the BEELINE framework [36], which includes 7 scRNA-seq datasets of 5 mouse and 2 human cell lines (see Methods; Additional file 2: Table S1). For each dataset, three distinct types of ground-truth networks, including cell type-specific ChIP-seq, non-specific ChIP-seq, and functional interaction networks from the STRING database [38], were collected. In addition, a loss-of-function/gain-of-function (LOF/GOF) network was collected from the mouse embryonic stem cell (mESC) dataset [39]. The cell type-specific knowledge graphs for each dataset were constructed. Our results showed the gene–gene interactions in the knowledge graphs had little overlap with the ground truths, ranging from 0.133 to 2.853%, indicating the minimized risk of data leakage (Additional file 1: Fig. S1). Then, we compared the KEGNI and MAE with 8 established methods, including PIDC [8], GENIE3 [10], GRNBoost2 [11], scGeneRAI [12], AttentionGRN [16], SCODE [40], PPCOR [41], and SINCERITIES [42]. The performance was estimated based on early precision ratio (EPR), which was defined as the fraction of true positives among the top-k predicted edges compared to a random predictor. The *k* indicates the number of edges in the ground truth network (see Methods).

**Fig. 2:**
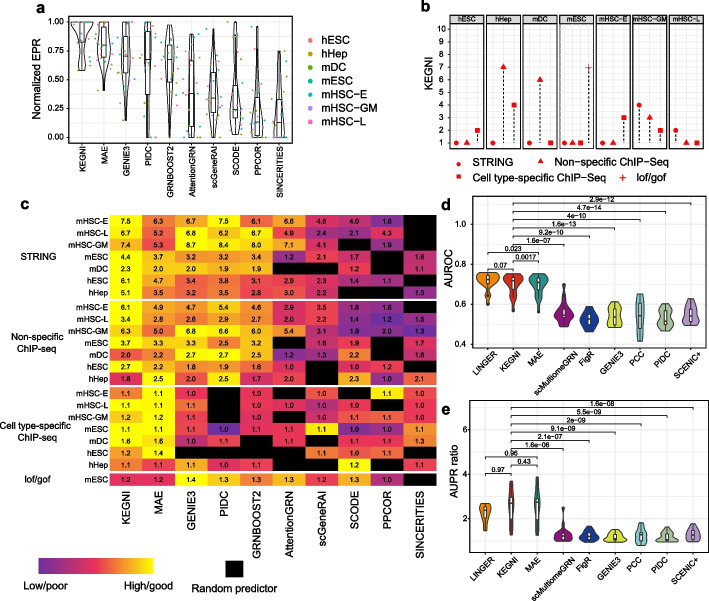
Performance evaluation of KEGNI and other methods in GRN inference. **a** Violin plots of normalized EPR values across methods. Colors represent distinct datasets, including human embryonic stem cells (hESC), human mature hepatocytes (hHep), mouse dendritic cells (mDC), mouse embryonic stem cells (mESC), and three lineages of mouse hematopoietic stem cells (mHSC), namely erythroid lineage (mHSC-E), granulocyte–macrophage lineage (mHSC-GM), and lymphoid lineage (mHSC-L). **b** Comparison of KEGNI rankings with other methods across all datasets and their corresponding ground truths. Rankings are visualized using different shapes, reflecting the sources of ground-truth networks: circles represent functional interaction networks from the STRING database, triangles denote non-specific ChIP-seq networks, squares indicate cell type-specific ChIP-seq networks, and plus signs (+) represent loss-of-function/gain-of-function networks. **c** Detailed EPR results for each dataset. Each row represents a scRNA-seq dataset, with colors scaled between 0 and 1 using the minimum–maximum scaling on EPR values. Black squares indicate cases where performance is lower than a random predictor. **d, e** Violin plots displaying the AUROC and AUPR ratio values of potential target genes across various TFs and cell types. *P* values were calculated using one-sided paired *t*-tests

To assess the stability and reproducibility of the KEGNI and MAE models, the median values from ten independent runs were used for comparison (Additional file 2: Table S2-S4). Our data demonstrated that the KEGNI framework achieved the best performance (Fig. 2a). Additionally, the MAE model outperformed other methods except KEGNI, suggesting that the self-supervised learning strategy effectively captures gene relationships from single-cell RNA-seq data. In detail, the KEGNI framework achieved the best performance across 12 benchmarks (Fig. 2b). Additionally, the MAE model excelled in four benchmarks, PIDC achieved the best performance on one benchmark, and GENIE3 achieved top results in 4 benchmarks (Additional file 1: Fig. S2). Notably, only KEGNI and MAE consistently outperformed random predictors across all benchmarks (Fig. 2c). We further compared KEGNI with SCENIC [9], which constructs a co-expression network using GENIE3 and prunes edges via RcisTarget [9, 43]. Evaluation using EPR shows that SCENIC outperforms both KEGNI and GENIE3 (Additional file 1: Fig. S3a). To ensure a fairer comparison, we applied RcisTarget in KEGNI to generate a variant termed KEGNI*, which outperforms SCENIC on the EPR metric (Additional file 1: Fig. S3a). To further evaluate the coverage of regulatory interactions, we assessed the performance using the area under the precision-recall curve (AUPR). Our results indicate that while edge pruning improves precision, it may also increase false negatives (Additional file 1: Fig. S3b), suggesting the need to balance precision and recall when applying filtering strategies. Moreover, when evaluating algorithms on datasets consisting of all significantly varying TFs and the 1000 most variable genes, KEGNI and MAE continued to demonstrate superior performance compared to other algorithms (Additional file 1: Figs. S4 and S5).

To evaluate the robustness of KEGNI, we performed a sensitivity analysis on hyperparameters, including the number of neighbors in the k-NN algorithm and the balancing coefficient between the MAE loss and KGE loss (see Methods, Additional file 2: Tables S5 and Table S6). The analysis was performed on datasets comprising all significantly varying TFs and the 500 most variable genes. The results indicate that KEGNI achieves stable and good performance under the default parameter settings. Overall, the comparison using the BEELINE framework confirms the consistent and reliable superiority of the KEGNI and MAE models, suggesting that the self-supervised learning approach is particularly effective for GRN inference tasks, and that incorporating high-quality prior knowledge can further enhance the performance.

To fully demonstrate the effectiveness of KEGNI and MAE, we benchmarked them against LINGER [22], SCENIC + [21], scMultiomeGRN [23], and FigR [20], all of which leverage scRNA-seq and scATAC-seq datasets simultaneously. Additionally, GENIE3 [10], PIDC [8], and Pearson’s correlation coefficient (PCC) methods that only use scRNA-seq data were included in comparison. As described in the LINGER [22], we used public peripheral blood mononuclear cells (PBMCs) from 10 × Genomics as inputs and employed putative targets of TFs from 20 blood cell chromatin immunoprecipitation followed by sequencing (ChIP-seq) datasets as ground truths.

For each ground truth, the area under the receiver operating characteristic curve (AUROC) and AUPR ratios were calculated. Overall, KEGNI, MAE, and LINGER outperformed other tools, exhibiting significantly higher AUROC (Fig. 2d and Additional file 2: Table S7) and AUPR ratios (Fig. 2e and Additional file 2: Table S8). Specifically, the AUROC of LINGER and KEGNI significantly surpassed MAE. The performances of LINGER (average AUROC = 0.714) and KEGNI (average AUROC = 0.699) were comparable. Regarding the AUPR ratio, no significant differences in AUPR performance were observed among KEGNI, MAE, and LINGER. In summary, the comprehensive analysis highlights KEGNI’s effectiveness for GRN inference from scRNA-seq data and easily accessible knowledge, and underscores its competitive performance compared to tools that utilize paired scRNA-seq and scATAC-seq data.

### Investigating the biological meaning of latent representations

**Fig. 3:**
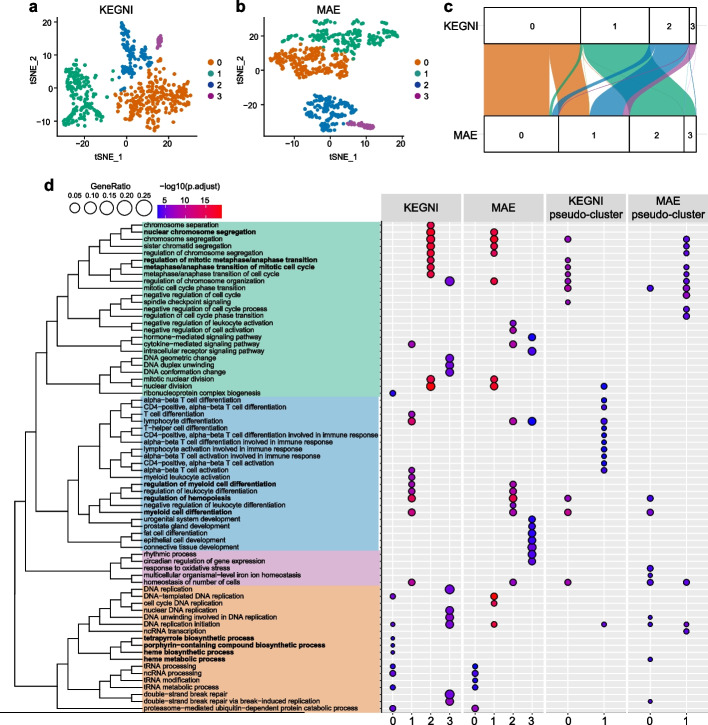
Investigating the biological meaning of latent representations produced by KEGNI and MAE. **a, b** t-Distributed Stochastic Neighbor Embedding (t-SNE) visualization of latent representations generated by KEGNI (**a**) and MAE (**b**), with gene representations clustered using the Louvain method. **c** Sankey plot illustrating gene flow between clusters identified in **a** and **b**, with flow width indicating gene abundance. **d** GO analysis across clusters of KEGNI, MAE, and their pseudo-clusters. *P*-values were calculated using one-sided Fisher’s exact test and adjusted for multiple comparisons with the Benjamini–Hochberg false discovery rate method. GO terms in bold are associated with the intrinsic features of the mHSC-E cell type

After demonstrating the superior performance of KEGNI and MAE in GRN inference, we conducted further investigations to uncover the underlying reasons for their remarkable results. We initiated this exploration by analyzing the latent space constructed by KEGNI and MAE using the mHSC-E [44] dataset, which has been described in the BEELINE framework [36]. Assuming that related genes should be clustered closer together in the latent space, we applied the Louvain method to these gene representations and clustered the genes into four distinct groups. These clusters were visualized using t-Distributed Stochastic Neighbor Embedding (t-SNE) (Fig. 3a and 3b). Divergence in gene flow among the clustering results is illustrated in the Sankey plot (Fig. 3c).

To better understand the functional roles of genes within distinct clusters, we performed Gene Ontology (GO) enrichment analysis. To account for potential randomness, we also randomly assigned genes into four clusters, ensuring that the number of genes in each cluster matched those in the clusters from KEGNI and MAE. These artificially generated clusters, referred to as “KEGNI pseudo-cluster” and “MAE pseudo-cluster,” were also subjected to GO analysis. The top 10 enriched biological process GO terms from each cluster were chosen for visualization and comparison (Fig. 3d and Additional file 2: Table S9). The semantic similarity of these GO terms was measured using GOSemSim [45, 46], and the terms were categorized into four distinct groups via hierarchical clustering. Notably, GO terms related to the mitotic cell cycle were highly represented across clustering results from KEGNI, MAE, and even the pseudo-clustering analysis. This observation aligns with the original study of mHSC-E [44], which highlights the critical role of the mitotic cell cycle in erythrocyte development. Specifically, the KEGNI_1 and MAE_2 clusters showed significant enrichment in GO terms related to intrinsic features of the mHSC, such as “regulation of myeloid cell differentiation,” whereas these terms were absent in the pseudo-cluster results. Additionally, the KEGNI_0 cluster was notably enriched in GO terms related to the “tetrapyrrole biosynthetic process” and “porphyrin-containing compound biosynthetic process,” which is also consistent with findings reported in previous research [44]. These results demonstrated the gene embeddings generated by KEGNI and MAE captured biologically relevant patterns. Furthermore, to quantify the clustering performance of KEGNI and MAE, we calculated the Adjusted Rand Index (ARI) using labels derived from the hierarchical clustering of GO terms as reference. The clusters generated by KEGNI demonstrated a higher ARI (0.575) compared to those generated by MAE (0.407), indicating that KEGNI provides more biologically meaningful and interpretable representations.

### Validation of GRN prediction in ChIP-seq and perturbation dataset

**Fig. 4:**
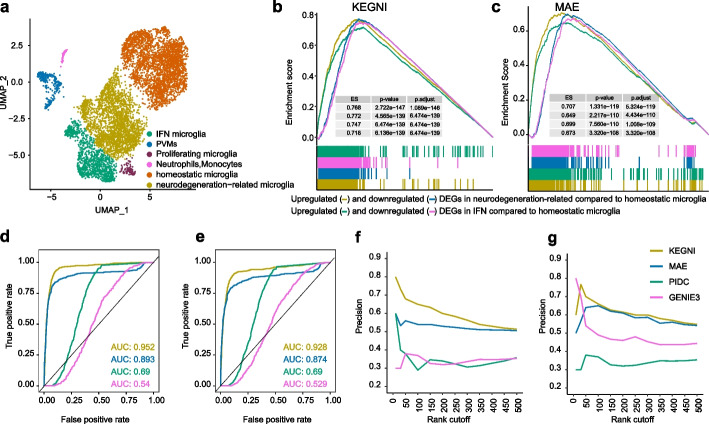
Evaluation of GRN prediction on microglia dataset with perturbation and ChIP-seq data. **a** UMAP plot of microglia cells displaying the distribution of *Cop1*-KO and WT cells across six distinct clusters. **b, c** Genes regulated by *Cop1* were ranked by edge weight from KEGNI (**b**) and MAE (**c**) for GSEA. *P*-values of enrichment scores (ES) were calculated using permutation tests and adjusted for multiple comparisons with the Benjamini–Hochberg false discovery rate method. **d, e** ROC curves for *Cop1*-regulated genes predicted by KEGNI, MAE, PIDC, and GENIE3. The ground truths used for **d** and **e** are the sets of DEGs between neurodegeneration-related microglia and homeostatic microglia, and DEGs between IFN microglia and homeostatic microglia, separately. **f, g** Precision curves of the top-k predicted genes regulated by *Cebpb* in neurodegeneration-related microglia (**f**) and IFN microglia (**g**), with the ground truth derived from ChIP-seq data of c/EBPβ targets in *Cop1*-KO primary microglia. Results in **f** and **g** are shown for *k* values ranging from 1 to 500

To further assess the capabilities of KEGNI, we utilized scRNA-seq and ChIP-seq datasets from a study examining the role of ubiquitin ligase COP1 in the post-translational modification of c/EBPβ and regulation of pro-inflammatory gene expression in microglia [47]. We analyzed the scRNA-seq data and identified 6 clusters in the microglia from *Cop1* knockout and control mice (Fig. 4a, Additional file 2: Table S1). Initially, we extracted the gene expression profile of homeostatic microglia cells as input for GRN inference. The differentially expressed genes (DEGs) between neurodegeneration-related microglia, IFN microglia from the *Cop1*-KO mice and wild-type homeostatic microglia were used as separate ground truths (Additional file 2: Tables S10 and S11). The genes regulated by *Cop1* in the GRN were selected and ranked according to their edge weights. We conducted Gene Set Enrichment Analysis (GSEA) and calculated AUROC to evaluate the performance of KEGNI and MAE in comparison to GENIE3 and PIDC using the ranked gene list. The results indicated that KEGNI consistently achieved higher Enrichment Score (ES) and significantly lower adjusted *p*-values for all ground-truth gene sets compared to MAE, whereas predictions from GENIE3 and PIDC did not demonstrate significant enrichment (Fig. 4b, c). In terms of AUROC, our analysis revealed that KEGNI achieved scores of 0.952 and 0.928 for the two respective ground truths, compared to 0.893 and 0.874 for MAE, 0.690 and 0.690 for PIDC, and 0.540 and 0.529 for GENIE3 (Fig. 4d, e).

Then, we used gene expression profiles of neurodegeneration-related microglia and IFN microglia cells as inputs for GRN inference. We prepared the ground truth from ChIP-seq of c/EBPβ in *Cop1*-KO primary microglia and employed ChIPseeker [48] to annotate peaks, adopting the nearest annotated genes as the ground truth for c/EBPβ binding sites. Similarly, genes regulated by *Cebpb* were ranked based on their edge weights. We calculated the precision of the top-k predictions to quantify the performance of KEGNI, MAE, GENIE3, and PIDC. KEGNI achieved superior performance over other methods (Fig. 4f, g). Additionally, our analysis indicates that highest-confidence target genes with higher edge weights are more likely to be confirmed by the ground truth, highlighting the importance of edge weights in GRN inference. Overall, the comparison results using both perturbation data and ChIP-seq data from the microglia datasets reaffirm that KEGNI outperforms existing methods in GRN inference. Moreover, the evaluation using DEGs between *Cop1*-KO and WT cell subpopulations as ground truths further demonstrates KEGNI’s effectiveness in predicting non-TF interactions.

### Identification of driver genes in regulatory network

**Fig. 5:**
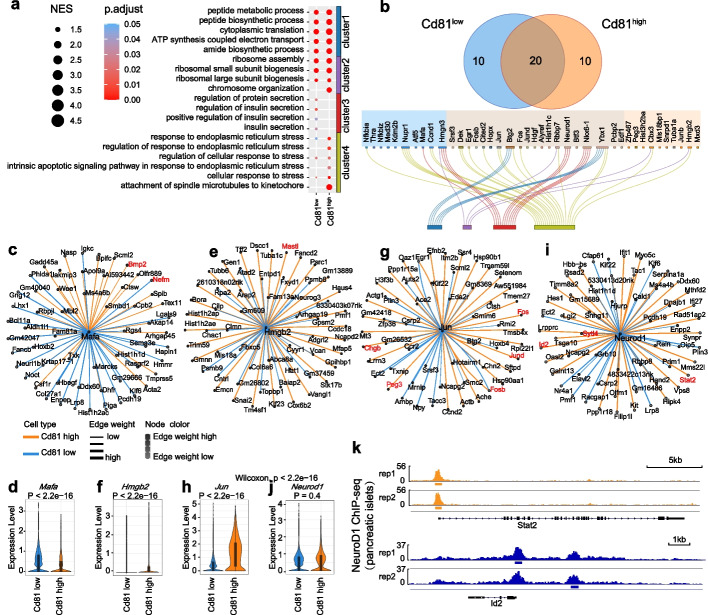
Driver genes identification and network comparison. **a** GSEA results for driver genes identified in *Cd81* ^low^ and *Cd81* ^high^ subpopulations. *P*-values for enrichment score (ES) were calculated using permutation tests and adjusted for multiple comparisons with the Benjamini–Hochberg false discovery rate method. **b** Venn diagram depicting the top 30 predicted driver TFs shared or unique to each subpopulation. **c, e, g, i** Subnetworks of key driver genes, including *Mafa* (**c**), *Hmgb2* (**e**), *Jun* (**g**), and *Neurod1* (**i**). Nodes represent genes, and edges are colored yellow for *Cd81* ^high^ and blue for *Cd81* ^low^, with edge thickness corresponding to edge weight. **d, f, h, j** Violin plots showing expression levels of *Mafa* (**d**), *Hmgb2* (**f**), *Jun* (**h**), and *Neurod1* (**j**) in *Cd81* ^high^ and *Cd81* ^low^ subpopulations. *P*-values were calculated using an unpaired Wilcoxon rank-sum test. **k** Integrated Genomics Viewer (IGV) snapshots showing ChIP-seq data for NeuroD1, displaying regulatory interactions with *Stat2* and *Id2* in pancreatic islets

To evaluate the performance of KEGNI in the identification of driver genes in regulatory networks, we conducted a comparative analysis of GRNs using a dataset of pancreatic beta-cell from high-fat diet (HFD)-fed mice (Additional file 2: Table S1) [49]. We introduced a regulation score based on the edge weight to quantify the regulatory potential of genes within the constructed GRN. The driver genes were defined by high cumulative regulation scores across target genes. To identify the driver genes and their functional roles in the *Cd81* ^low^ and *Cd81* ^high^ subpopulations of β-cells, which were characterized by stress-related and inflammatory pathways enrichment, we performed GSEA using gene sets from gene ontology. The significantly enriched GO terms were categorized into four distinct clusters based on their semantic similarity (Fig. 5a and Additional file 2: Table S12). Notably, the *Cd81* ^low^ subpopulation exhibited enrichment in the cluster of insulin secretion regulation, whereas the *Cd81* ^high^ subpopulation exhibited enrichment in the cluster of response to endoplasmic reticulum stress. These results showed the distinct regulatory mechanisms and functional states of the *Cd81* ^low^ and *Cd81* ^high^ subpopulations. Then, we identified top-ranked driver TFs in the *Cd81* ^low^ and *Cd81* ^high^ subpopulations according to the regulation score (Fig. 5b). Majority of these TFs were Linked to the 4 clusters of GO terms (Additional file 2: Table S13).

Furthermore, the divergent regulatory networks formed by driver TFs and their target genes were analyzed. Mafa is a unique driver TF and highly expressed in the *Cd81* ^low^ subpopulation as a key regulator of mature β-cell. Mafa exhibited a high regulation score with a β-cell maturity marker *Nefm* [50] in the *Cd81* ^low^ subpopulation and had a high regulation score with inflammatory factor Bmp2 in the *Cd81* ^high^ subpopulation (Fig. 5c, d). The expression of Bmp2 could lead to a loss of β-cell maturity via inhibition of NeuroD1 activity and downregulation of *Mafa* [51, 52]. Additionally, NF-κB inhibitors IκBα (*Nfkbia*) and IκBζ (*Nfkbiz*) were identified as unique driver TFs in the *Cd81* ^low^ subpopulation (Fig. 5b) [53–55]. We also identified the pro-inflammatory factor Hmgb2 as a driver TF in the *Cd81* ^high^ subpopulation. Hmgb2, which had been shown to mediate various inflammatory diseases [56–58], exhibited a high regulation score with inflammatory cytokine-related *Mastl* [59] (Fig. 5e, f). The shared driver TF Jun in the two subpopulations exhibited high regulation scores with *Fos*, *Fosb*, *Jund*, and *Chgb* [49] (Fig. 5g, h). The stress-responsive factors Fos and FosB can form heterodimers with Jun proteins to regulate gene expression in β-cells [60, 61]. These interactions were also observed in the subnetwork of Jund, Fos, and Fosb (Additional file 1: Figs. S6 and S7). Moreover, the critical TF NeuroD1 in β-cell maturation and maintenance [51] was a driver TF in both subpopulations (Fig. 5i, j). In the *Cd81* ^high^ subpopulation, *Neurod1* displayed a high regulation score with stress-related genes including *Stat2* and *Id2*. STAT2 plays a role in regulating apoptosis and inflammation [62]. ID2, a DNA-binding protein inhibitor, has been shown to prevent NeuroD1 from binding to its DNA targets [63]. To validate these predictions, we collected ChIP-seq data for NeuroD1 in pancreatic islets [52] and identified NeuroD1 binding sites in the promoter regions of both *Stat2* and *Id2*, further demonstrating KEGNI’s ability to accurately identify direct gene targets (Fig. 5k). Altogether, these findings revealed the potential important functions of driver TFs in β-cell subpopulations. The identified driver TFs exhibit diverse functions and regulatory roles across different conditions based on GRNs inferred by the KEGNI framework. Overall, these findings underscore KEGNI’s ability to reveal the intricate regulatory mechanisms and gene networks shaped by specific conditions and cellular contexts.

## Discussion

Leveraging a self-supervised learning model, KEGNI effectively captures intrinsic information within scRNA-seq data with a knowledge graph-guided training strategy for inferring GRNs. Our assessments based on the BEELINE benchmark demonstrated that KEGNI can effectively achieve superior performance in comprehensive GRN inference tasks. KEGNI exhibited superior performance in predicting interactions among non-TFs and direct regulations, and showed competitive performance compared to the tools integrating paired scRNA-seq and scATAC-seq data. As a generalizable framework, the KGE and MAE models can be configured independently. The MAE module based on scRNA-seq data only could offer a more broadly applicable solution for GRN inference. The scalable knowledge graphs in KEGNI, which can incorporate diverse regulatory networks that encompass various node types such as miRNA and lncRNA, are not limited to current guidance knowledge databases in the KEGNI.

The limitations of KEGNI include its reliance on cell marker database and the lack of both direct and causal targets analysis in the GRNs. The cell markers used for constructing cell type-specific knowledge graphs in KEGNI are sourced from established databases, which may not completely align with specific datasets. The selection of markers tailored to the dataset could be a crucial step in constructing knowledge graphs (Additional file 1: Supplementary Notes 1 and 2). Moreover, the GRNs constructed by KEGNI do not indicate direct regulation relationships. Methods that leverage both scATAC-seq and scRNA-seq enable the modeling of cis-regulatory elements (RE), allowing the prediction of cis-regulation (RE-TG) and TF-binding (TF-RE) relationships [21, 22], which is not feasible for KEGNI using only scRNA-seq data. The GRNs could be refined by removing indirect targets through cis-regulatory motif analysis [9, 43, 64] or deep learning methods [65], thereby allowing high-confidence prediction of key regulators and their direct target genes (Additional file 1: Fig. S3). In addition, integrating time-series or pseudotime data can help reveal causal regulatory events and dynamic GRNs [17, 66–70], which is not included in KEGNI due to challenges in obtaining and integrating accurate temporal data with graph neural networks [36, 68, 71, 72]. It remains a promising research direction in the future.

As a general self-supervised learning framework, KEGNI effectively integrates scRNA-seq data with prior knowledge for inferring cell type-specific regulatory networks. The GRNs constructed by KEGNI highlight the framework’s capability to elucidate complex regulatory events and gene expression changes in cells.

## Conclusions

Here, we present KEGNI (Knowledge graph-Enhanced Gene regulatory Network Inference), a knowledge-guided framework for inferring cell type-specific gene regulatory networks (GRNs) from scRNA-seq data by integrating prior biological knowledge. KEGNI employs a graph autoencoder to capture regulatory relationships from gene expression profiles and incorporates a knowledge graph to enhance biological relevance, addressing limitations of purely data-driven approaches that rely on sparse or unpaired multi-omics data. KEGNI outperforms existing scRNA-seq-based methods in accuracy and robustness, as demonstrated by extensive evaluations on diverse ground-truth datasets. It achieves competitive performance to methods requiring paired scRNA-seq and scATAC-seq data, even without relying on additional epigenetic data. When applied to pancreas islet datasets, KEGNI successfully identifies key driver genes and reveals context-specific regulatory mechanisms, demonstrating its ability to uncover biologically meaningful interactions. Given the critical role of gene regulation in cellular function and disease, we expect KEGNI will help researchers construct more accurate cell type-specific GRNs, offering deeper insights into gene regulation and its complex roles in biological systems.

## Methods

### Cell type-specific GRN inference

KEGNI is an end-to-end framework designed for cell type-specific GRN inference. The framework requires two inputs: a single-cell expression matrix annotated with cell type information and a set of cell type-specific marker genes. The output of KEGNI is a weight matrix that quantifies the regulatory interactions between genes, which can be used to construct GRNs and identify potential driver genes.

### Base GRN construction and gene representation

Based on the gene expression matrix, an initial GRN is constructed using *k* nearest neighbors (k-NN) algorithm with Euclidean distance as the similarity metric. This network is represented as an unweighted graph $G=\left(V,A,X\right)$, where $V={\{v}_{1},{v}_{2},\dots ,{v}_{N}\}$ denotes the set of nodes, with each ${v}_{i}$ representing a gene; $\text{A}\in {\{0,1\}}^{N\times N}$ is the adjacency matrix; and $X=\left[{x}_{1},{x}_{2},\dots ,{x}_{N}\right]\in {\mathbb{R}}^{N\times d}$ is the node feature matrix, with each ${x}_{i}$ corresponding to the expression level of gene ${v}_{i}$.

The MAE model, a masked graph autoencoder, is used to learn the latent gene representation $H=\left[{h}_{1},{h}_{2},\dots ,{h}_{N}\right]\in {\mathbb{R}}^{N\times {d}_{h}}$, where ${h}_{i}\in {\mathbb{R}}^{{d}_{h}}$ represents the latent embedding of node $i$, and ${d}_{h}$ denotes the dimension of these embeddings. Both the encoder (${f}_{E}$) and decoder (${f}_{D}$) in the MAE model are composed of Graph Attention Network (GAT) layers. The hidden representation ${h}_{i}^{\left(l+1\right)}$ of node ${v}_{i}$ at layer $\left(l+1\right)$ is computed as follows:

1 $$\begin{array}{c}{h}_{i}^{\left(l+1\right)}=\sigma \left(\sum_{j\in {\mathcal{N}}_{i}}{\alpha }_{ij}^{\left(l\right)}{W}^{\left(l\right)}{h}_{j}^{\left(l\right)}\right)\end{array}$$

where $\sigma$ is an activation function, ${W}^{\left(l\right)}$ is a shared weight matrix, and ${\alpha }_{ij}^{\left(l\right)}$ is the attention coefficient determined by the attention mechanism. The attention coefficient is given by:

2 $$\begin{array}{c}{\alpha }_{ij}^{\left(l\right)}=\frac{\text{exp}\left({\text{LeakyReLU}}\left({\text{a}}^{T}\left[{W}^{\left(l\right)}{h}_{i}^{\left(l\right)}\parallel {W}^{\left(l\right)}{h}_{j}^{\left(l\right)}\right]\right)\right)}{\sum_{k\in {\mathcal{N}}_{\text{i}}}\text{exp}\left({\text{LeakyReLU}}\left({\text{a}}^{T}\left[{W}^{\left(l\right)}{h}_{i}^{\left(l\right)}\parallel {W}^{\left(l\right)}{h}_{k}^{\left(l\right)}\right]\right)\right)}\end{array}$$

Here, $\mathbf{a}$ is a learnable weight vector, $||$ denotes the concatenation operation, and ${\mathcal{N}}_{i}$ represents the set of neighbors of node ${v}_{i}$.

Inspired by GraphMAE [35], the MAE model employed a self-supervised learning strategy to learn meaningful gene representations. Specifically, a masked node feature matrix ${X}_{\text{masked}}$ is generated by randomly replacing the features of a subset of nodes $\widetilde{V}\subset V$ with a special mask token. Given the masked feature matrix ${X}_{\text{masked}}$ and the adjacency matrix $A$, the objective of the MAE model is to reconstruct the original features $\widetilde{X}$ of the masked nodes.

3 $$\begin{array}{c}H={f}_{E}\left({X}_{masked},A\right),\widetilde{X}={f}_{D}\left(H,A\right)\end{array}$$

The scaled cosine error (SCE) loss function is used to assess the quality of reconstruction, as previously reported [35]. The SCE loss function is defined as:

4 $$\begin{array}{c}{\mathcal{L}}_{\text{MAE}}=\frac{1}{\left|\widetilde{\mathcal{V}}\right|}{{\sum }_{{v}_{i}\in \widetilde{\mathcal{V}}}\left(1-\frac{{{x}_{i}}^{T}{\widetilde{x}}_{i}}{\left|\left|{x}_{i}\right|\right|\cdot \left|\left|{\widetilde{x}}_{i}\right|\right|}\right)}^{\gamma }, \gamma \ge 1)\end{array}$$

where ${x}_{i}$ is the original feature vector of node ${v}_{i}$, ${\widetilde{x}}_{i}$ is the corresponding reconstructed feature vector from $\widetilde{X}$, $\widetilde{\mathcal{V}}$ is the set of masked nodes, and $\upgamma$ is a scaling factor used to control the weighting of different samples, with a default value of 1, which implies equal weighting of all sample errors during training. The SCE loss is averaged over all masked nodes, providing a measure of how closely the reconstructed features match the original masked features.

### Knowledge graph construction and representation

We constructed the knowledge graph based on the KEGG pathway database, which contain manually curated and continuously updated information about molecular interactions. Description and comparison of different databases used for knowledge graph construction can be found in Additional file 1: Supplementary Note 1 and Additional file 2: Table S14. To construct the knowledge graph, we downloaded KGML files from the KEGG PATHWAY database (https://www.kegg.jp/kegg/pathway.html). We processed these files using the R package KEGGgraph (v.1.62.0) [73]. We focused on two types of relations: “PPrel” for protein–protein interactions and “Gerel” for gene expression interactions. More specifically, there are 14 relation types between genes or proteins, and we concentrated on direct gene relationships (“activation,” “inhibition,” “expression,” “repression,” “dephosphorylation,”

“phosphorylation,” “glycosylation,” “ubiquitination,” and “methylation,” while excluding “indirect effect,” “state change,” “binding/association,” “dissociation,” and “missing interaction”). To reduce the complexity of model training, we categorized “activation” and “expression” as “positive effects,” “inhibition” and “repression” as “negative effects,” and “dephosphorylation,” “phosphorylation,” “glycosylation,” “ubiquitination,” and “methylation” as “uncertain effects.”

We organized the filtered gene regulatory information into a base knowledge graph. Subsequently, according to cell type annotations, we retrieved cell type-specific markers from the CellMarker 2.0 database (http://bio-bigdata.hrbmu.edu.cn/CellMarker/). Utilizing these markers, we refined the base knowledge graph into a cell type-specific knowledge graph by retaining nodes corresponding to the cell type marker genes and including their first-order neighbors (genes directly connected to the marker genes). More details about the construction of the cell type-specific knowledge graph using cell type-specific markers are provided in Additional file 1: Supplementary Note 2. The basic unit of this knowledge graph is represented as a triplet $\left(h,r,t\right)$, where $h$ (head entity) and $t$ (tail entity) are entities, including genes or proteins, and $r$ (relation) denotes the relationship connecting these entities. To learn representations of entities and relations in the knowledge graph, the KGE (Knowledge Graph Embedding) model employs a contrastive learning strategy with negative sampling. The loss function of the KGE model is defined as:

5 $$\begin{array}{c}{\mathcal{L}}_{KGE}=-log\sigma \left(d\left(h,t\right)\right)-\sum_{i=1}^{n}\frac{1}{n}log\sigma \left(-d\left(\left\{{h}_{i}^{{{\prime}}}\right\},\left\{{t}_{i}^{{{\prime}}}\right\}\right)\right)\end{array}$$

Here, $\left({\text{h}}_{\text{i}}^{{{\prime}}},{\text{t}}_{\text{i}}^{{{\prime}}}\right)$ represents the negative samples, where head or tail entities are randomly sampled to construct corrupted triples. $n$ is the number of negative samples, $\sigma$ is the sigmoid function, and $d$ is the scoring function. We tested both ComplEx [74] and TransE [75, 76] as knowledge graph embedding methods, and the results showed that ComplEx outperforms TransE (see Additional file 2: Table S15 and Additional file 1: Supplementary Note 3). The superior performance of ComplEx may be due to its better ability to model antisymmetric and one-to-many relational patterns present in the knowledge graph [77]. Therefore, we adopted the scoring function of ComplEx:

6 $$\begin{array}{c}{d}_{r}\left(h,t\right)={\text{R}}{\text{e}}\left({h}^{{\top }}diag\left(r\right)\bar{t}\right)\end{array}$$

In this equation, $\text{Re}$ denotes the real part of a complex number, and $\overline{t}$ represents the complex conjugate of the tail entity’s embedding $t$. The term $diag\left(r\right)$ represents a diagonal matrix whose diagonal entries are the elements of the complex-valued relation embedding vector $r$. The KGE model aims to maximize the score for true triples while minimizing the score for negative triples. We define the sets of triples and entities as $T$ and $E$, respectively. Entities are classified into two distinct categories: ${E}_{\text{scg}}$ for genes derived from single-cell expression profiles and ${E}_{\text{kgg}}$ for genes exclusive to the cell type-specific knowledge graph. When both the head and tail entities of a triple are genes from ${E}_{\text{scg}}$, the triple is denoted as ${T}_{\text{scg-scg}}$. Conversely, if the tail entity is a gene from ${E}_{\text{kgg}}$, the triple is denoted as ${T}_{\text{scg-kgg}}$.

For each positive triple $\left(h,r,t\right)\in T$, the corresponding negative triple set ${T}^{{{\prime}}}$ is generated by replacing either the head or the tail entity with another entity from the appropriate subset. For ${\text{T}}_{\text{scg}-\text{kgg}}^{{{\prime}}}$, we tested two strategies for generating negative samples: replacing both the head and tail entities or replacing only the tail entity. The results showed no significant performance differences between the two approaches. Therefore, we adopted the simpler strategy of replacing only the tail entity in our final implementation (Additional file 2: Table S16). Specifically, the strategy for generating negative samples is as follows:

7 $$\begin{array}{c}\begin{array}{c}{T}_{\text{scg}-\text{scg}}^{{{\prime}}}\left(h,r,t\right)=\{\left({h}^{{{\prime}}},r,t\right)\left|h^{{\prime}}\in {E}_{scg},t\in {E}_{scg}\}\cup \{\left(h,r,{t}^{{{\prime}}}\right)\right|h\in {E}_{scg},{t}^{{{\prime}}}\in {E}_{scg}\}\\ {T}_{scg-kgg}^{{{\prime}}}\left(h,r,t\right)=\{\left(h,r,{t}^{{{\prime}}}\right)|{t}^{{{\prime}}}\in {E}_{kgg}\}\end{array}\end{array}$$

In the case of ${\text{T}}_{scg-scg}^{{{\prime}}}$, negative samples are created by replacing either the head or tail entity with another entity from ${E}_{scg}$, while preserving the relation $r$. For ${\text{T}}_{\text{scg}-\text{kgg}}^{{{\prime}}}$ triples, only the tail entity is replaced with another entity from ${E}_{kgg}$, while keeping the head entity and the relation unchanged.

### Multi-task learning strategy

We utilized a multi-task learning strategy to simultaneously optimize the objectives of feature reconstruction and knowledge graph embedding. Initially, the MAE model was trained to learn gene embeddings based on gene expression data. For genes present in both the scRNA-seq data and the cell type-specific knowledge graph, the hidden embeddings learned by the MAE model were shared with the KGE model. For ${E}_{kgg}$ (genes exclusive to the cell type-specific knowledge graph), the embeddings are randomly initialized and updated during training. To ensure the compatibility of representations learned by the MAE model, we introduce an additional linear layer that projects the embeddings onto a shared and unified latent space before passing them to the KGE model. The unified objective of our model is defined as follows:

8 $$\begin{array}{c}{\mathcal{L}}_{\text{total}}={\mathcal{L}}_{\text{MAE}}+{ \lambda \mathcal{L}}_{\text{KGE}}\end{array}$$

Here, $\lambda$ is a hyperparameter that balances the contributions of the MAE and KGE losses. To assess the robustness of our model, we conducted a sensitivity analysis on $\lambda$ (Additional file 2: Table S6). The results indicate that setting $\lambda$ to 1 achieves good performance across most datasets. Finally, we used the transformed embeddings to quantify the regulatory potential between genes. Specifically, to ensure numerical stability, the hidden embeddings $h$ of the genes were first scaled to a range between − 1 and 1 using the $\text{tanh}$ function. The regulatory potential between genes was then calculated using the dot product.

### Datasets and ground truth

#### BEELINE dataset

The BEELINE [36] framework provides seven experimental single-cell RNA-seq datasets: (1) mESC: mouse embryonic stem cells (421 cells, 1120 genes in the TFs + 500 genes dataset, 1620 genes in the TFs + 1000 genes dataset) [39], (2) mDC: mouse dendritic cells (383 cells, 821 genes in the TFs + 500 genes dataset, 1321 genes in the TFs + 1000 genes dataset) [78], (3) three lineages of mouse hematopoietic stem cells [44], including mHSC-E: erythroid lineage (1071 cells, 704 genes in the TFs + 500 genes dataset, 1204 genes in the TFs + 1000 genes dataset), mHSC-GM: granulocyte–macrophage lineage (889 cells, 632 genes in the TFs + 500 genes dataset, 1132 genes in the TFs + 1000 genes dataset) and mHSC-L: lymphoid lineage (847 cells, 560 genes in the TFs + 500 genes dataset, 692 genes in the TFs + 1000 genes dataset), (4) hHep: human mature hepatocytes (425 cells, 948 genes in the TFs + 500 genes dataset, 1448 genes in the TFs + 1000 genes dataset) [79], and (5) hESC: human embryonic stem cells (758 cells, 910 genes in the TFs + 500 genes dataset, 1410 genes in the TFs + 1000 genes dataset) [80].

For each dataset, BEELINE collected three different types of ground-truth networks: cell type-specific ChIP-seq networks from the ENCODE, ChIP-Atlas, and ESCAPE databases corresponding to the same or similar cell types; nonspecific ChIP-seq networks from the DoRothEA, RegNetwork, and TRRUST databases; and functional interaction networks from the STRING database. Additionally, for the mESC dataset, BEELINE also collected an additional loss-of-function/gain-of-function (lof/gof) ground-truth network from the ESCAPE database. All of these datasets and networks are available on Zenodo (https://zenodo.org/records/3701939).

### PBMC dataset

The PBMC data used in this study were sourced from the 10 × Genomics website (https://support.10xgenomics.com/single-cell-multiome-atac-gex/datasets). The processed paired scRNA-seq and scATAC-seq data used in this study were derived from LINGER [22] and are available for download at: https://drive.google.com/file/d/1jwRgRHPJrKABOk7wImKONTtUupV7yJ9b/view?usp=sharing. Following the description in LINGER, we processed the PBMC dataset and retained 25,485 genes. The ground truth of TF-target regulatory relationships were collected from CistromeDB [81]. Ground truth data are available only for classical monocytes (1848 cells), myeloid dendritic cells (232 cells), naive B cells (282 cells), and naive CD4 T cells (1373 cells), and the relevant scRNA-seq and scATAC-seq were extracted for these cell types. Paired scRNA-seq and scATAC-seq data were used as inputs for LINGER, SCENIC +, FigR and scMultiomeGRN. KEGNI, MAE, PIDC, GENIE3, and PCC utilized only scRNA-seq data.

### COP1 dataset

We obtained the microglia single-cell RNA-seq data from GEO under accession number GSE145454 and processed the dataset according to the methodology described in the referenced paper [47]. After performing quality control, we retained 6720 cells, which were then divided into six clusters. Given the relevance of microglial functions to neurodegenerative diseases, we selected WT cells from the Homeostatic microglia cluster, which represents the baseline state of microglia, and selected *Cop1*-KO cells from the Neurodegeneration-related microglia and IFN microglia clusters due to their association with neuroinflammatory responses and microglial activation, respectively.

To evaluate the performance of GRN prediction using Homeostatic microglia cells, we extracted genes regulated by *Cop1* and ranked them based on their edge weights. Differentially expressed genes (DEGs) between the Homeostatic microglia dataset and the other two datasets were identified using FindMarkers in Seurat (v.4.4.0) [82] (see Additional file 2: Table S10 and S11) and were used as the ground truth. The gene expression profiles consisting of the top 2000 most highly variable genes and the DEGs were then used as inputs for GRN inference. To assess the performance of GRN prediction using neurodegeneration-related microglia and IFN microglia cells, genes regulated by *Cebpb* were extracted and ranked according to their edge weights. To establish the ground truth, we downloaded peak files from GSE145454 and utilized ChIPseeker [48] for peak annotation. Specifically, if a peak overlapped with the promoter region, defined as − 1000 to 1000 bp around any TSS, it was annotated as the nearest gene. These annotated genes were used as the ground truth.

### Pancreas islet beta cells dataset

We downloaded the pancreas islet dataset from the GEO under accession number GSE203376. The single-cell RNA-seq raw data were processed following the methodologies outlined in the original publication [49]. Subsequently, beta-cells were extracted and re-clustered into nine clusters. These clusters, which exhibit varying expression levels of *Cd81*, can be further defined into two subpopulations: *Cd81* ^low^ (14,246 cells) and *Cd81* ^high^ (3570 cells). The expression profiles of top 2000 most highly variable genes and four genes used in downstream analysis were used as inputs.

### Metrics

#### EPR

The EPR measures the precision of the top-$k$ predictions made by a GRN inference method, where $k$ is equal to the total number of edges in the ground-truth network. Early precision is calculated as the fraction of true positive edges among the top-$k$ predictions. The EPR is then determined by comparing the early precision to that of a random predictor, which is the edge density of the ground-truth network.

#### AUROC

The Area Under the Receiver Operating Characteristic Curve (AUROC) is a scalar value summarizing the overall performance of the method across all possible thresholds. The ROC curve plots the True Positive Rate (TPR) against the False Positive Rate (FPR) at various thresholds. AUROC measures a predictor’s ability to distinguish between positive and negative samples, with higher values indicating better performance.

#### AUPR

Precision is defined as the fraction of true positive links among all links predicted as positive. To measure the accuracy of a predictor, the AUPR ratio is defined as the ratio of the AUPR of a method to that of a random predictor. For a random predictor, the AUPR equals the fraction of positive samples in the dataset. The AUPR ratio is defined as $\frac{\text{AUPR}}{\text{AUPR of a random predictor}}$, representing the fold change in the accuracy of a predictor compared to random prediction.

### Driver genes and network comparison

We introduced a regulatory score to assess the influence of genes within the GRN. The regulatory score quantifies the impact of a gene $g$ by summing the edge weights of its top 200 target genes, selected based on the highest edge weights. Genes with high regulatory scores are designated as driver genes. Similarly, driver TFs are defined as those with high regulatory scores.

### Latent representation visualization and clustering

We began by scaling the gene embeddings and then applied PCA to reduce their dimensionality. The gene embeddings were then clustered using the Louvain method. For visualization, t-SNE was employed to depict the clusters. GO enrichment analysis was carried out for the genes within each cluster. To assess the similarity of significantly enriched GO terms, we used a graph-based method for GO structure analysis [83]. The enriched GO terms were subsequently classified into four distinct categories through hierarchical clustering. To validate the clustering results, the adjusted Rand index (ARI) was calculated to evaluate the agreement between the clustering outcomes and the GO term categories, with the latter serving as the reference labels. The implementation for ARI calculation is available on GitHub at https://github.com/Lipxiao/KEGNI.

### GSEA

The GSEA was performed using ClutserProfiler [84]. GSEA calculates the enrichment score (ES) to measure how a gene set is overrepresented at the extremes of a ranked list. The normalized enrichment score (NES) adjusts the ES for gene set size by comparing it to a null distribution obtained through permutation testing. The *p*-value is determined based on the proportion of permutations where the ES is greater than or equal to the observed ES.

## Supplementary Information

Additional file 1: Supplementary Figures S1–S7 and Supplementary Notes 1–3

Additional file 2: Supplementary Tables S1–S16

## Data Availability

The datasets and networks from the BEELINE framework are available on Zenodo (https://zenodo.org/records/3701939) [85]. The microglia single-cell RNA-seq dataset was obtained from GEO under accession number GSE145454 [86], and the pancreas islet dataset was downloaded from GEO under accession number GSE203376 [87]. The processed datasets, knowledge graphs and cell type-specific markers used in this study have been uploaded and are accessible at the Zenodo repository (https://zenodo.org/records/15711227) [88]. The source code for KEGNI with detailed parameter settings and usage instructions under the MIT license can be accessed at https://github.com/Lipxiao/KEGNI [89] and https://zenodo.org/records/15720607 [90].
