# Supplementary material for: KEGNI: knowledge graph enhanced framework for gene regulatory network inference
Source: Genome Biol. 2025 Sep 22;26:294. doi: 10.1186/s13059-025-03780-7 (PMC12455831; doi:10.1186/s13059-025-03780-7)
Supplement: Supplementary file 1 — Additional file 1: Supplementary Figures S1–S7 and Supplementary Notes 1–3 [file 13059_2025_3780_MOESM1_ESM.docx]

**Additional file 1 for**

**KEGNI: knowledge graph enhanced framework for gene regulatory network inference**

**Supplementary Figures S1–S7 and Supplementary Notes 1–3**

Pengxiao Li^1,4^, Lin Li^1,4^, Jingminjie Nan^2^, Jiahuan Chen^2^, Jielin Sun^1,5^ and Yanan Cao^2,3,^^5🖂^

^1^Shanghai Center for Systems Biomedicine, Key Laboratory of Systems Biomedicine (Ministry of Education), Institute of Translational Medicine, Shanghai Jiao Tong University, Shanghai, China.

^2^Ruijin Yangtze River Delta Health Institute, Wuxi Branch of Ruijin Hospital, Ruijin Hospital, Shanghai Jiao Tong University School of Medicine, Shanghai, China.

^3^Department of Endocrine and Metabolic Diseases, Shanghai Institute of Endocrine and Metabolic Diseases, Ruijin Hospital, Shanghai Jiao Tong University School of Medicine, Shanghai, China.

^4^These authors contributed equally: Pengxiao Li, Lin Li.

^5^These authors jointly supervised the work: Jielin Sun, Yanan Cao.

^🖂^e-mail: [jlsun@sjtu.edu.cn](mailto:jlsun@sjtu.edu.cn); [caoyanan@vip.sina.com](mailto:caoyanan@vip.sina.com)

**Supplementary Figures**


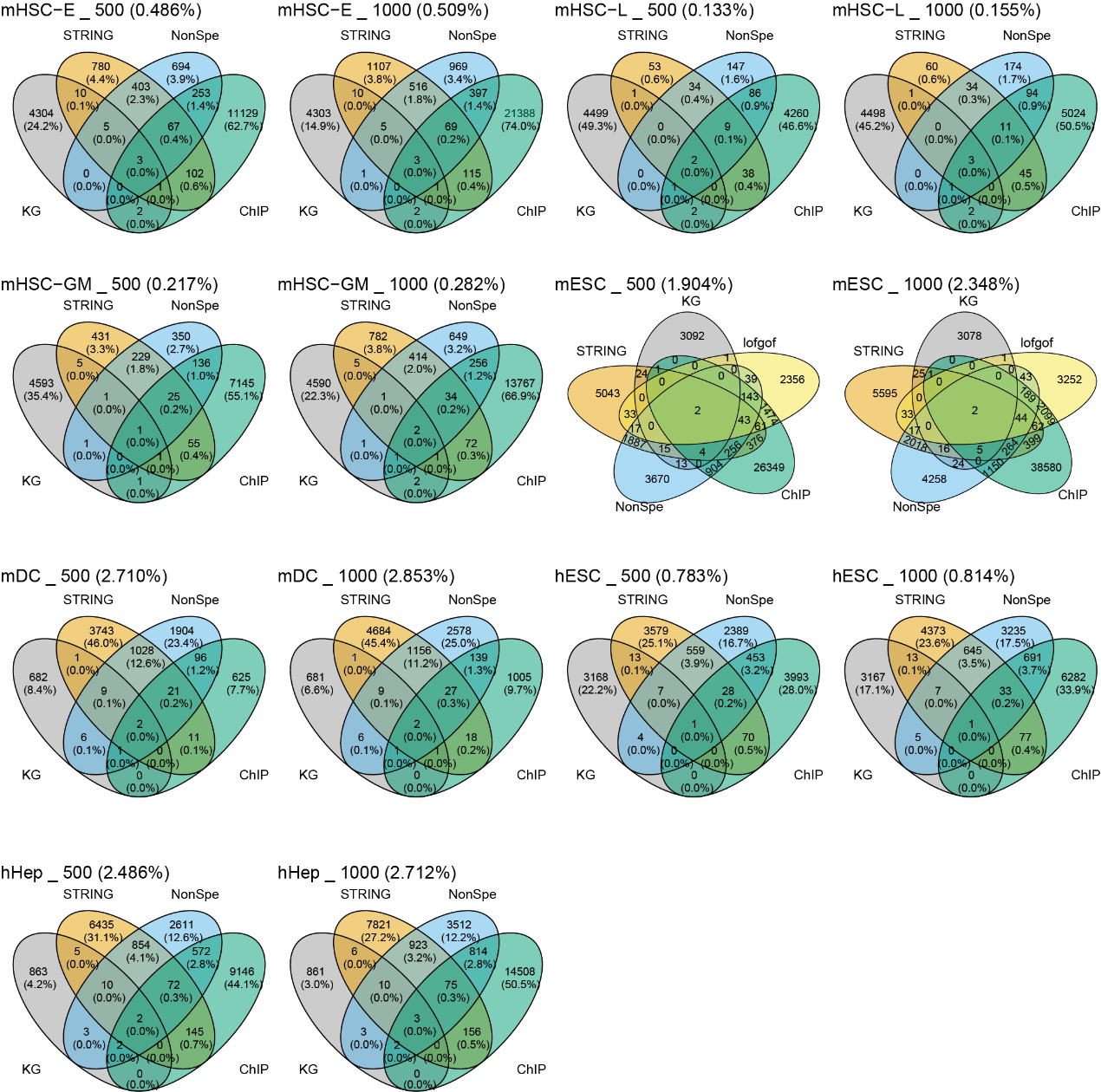


**Fig. S1.** Comparative Venn diagram showing gene-gene interactions between the cell type-specific knowledge graphs and ground truth data.


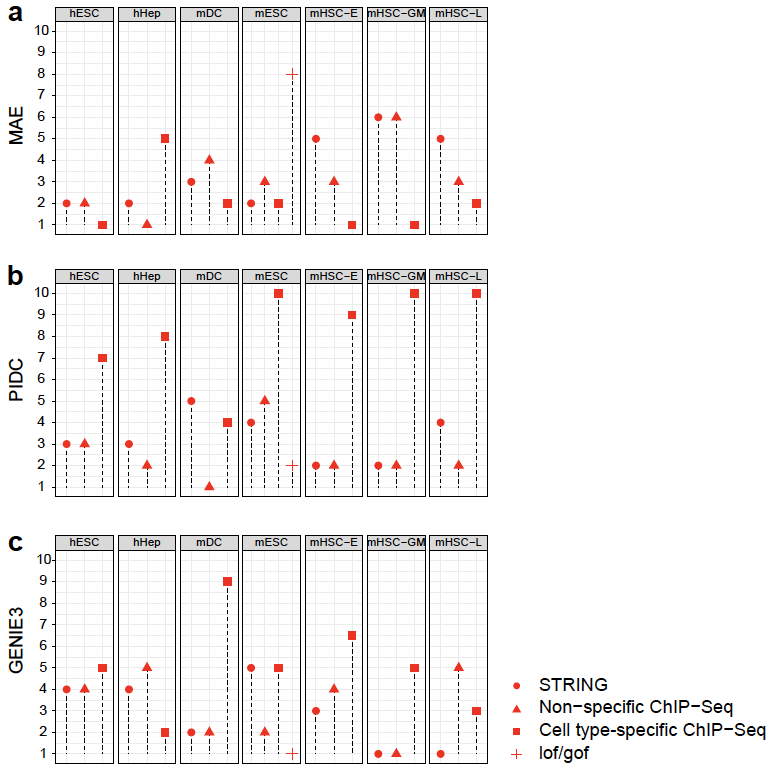


**Fig. S2.** Ranking results of MAE (**a**), PIDC (**b**), and GENIE3 (**c**) across all datasets with TFs and the 500 most variable genes. Rankings are visualized using different shapes, reflecting the sources of ground-truth networks: circles represent functional interaction networks from the STRING database, triangles denote non-specific ChIP-seq networks, squares indicate cell type-specific ChIP-seq networks, and plus signs (+) represent loss-of-function/gain-of-function networks.


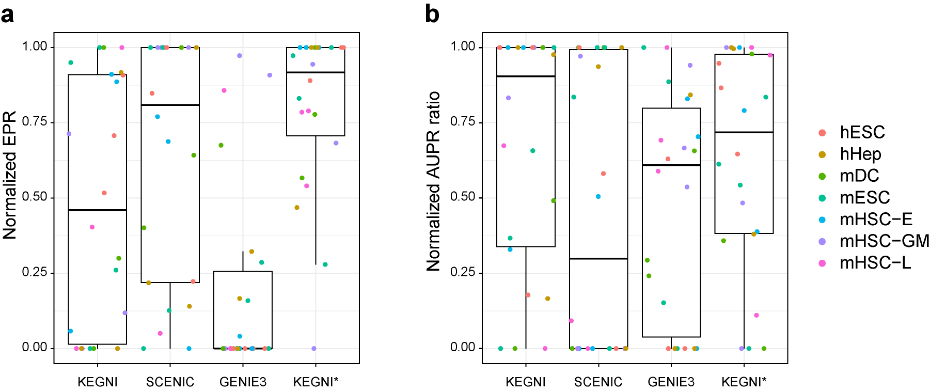


**Fig. S3.** Evaluation of the pruning strategy based on cis-regulatory motifs. **a** Normalized EPR of four methods across different benchmarks. **b** Normalized AUPR ratio of four methods across different benchmarks. KEGNI* denotes KEGNI incorporating motif-based filtering using RcisTarget.


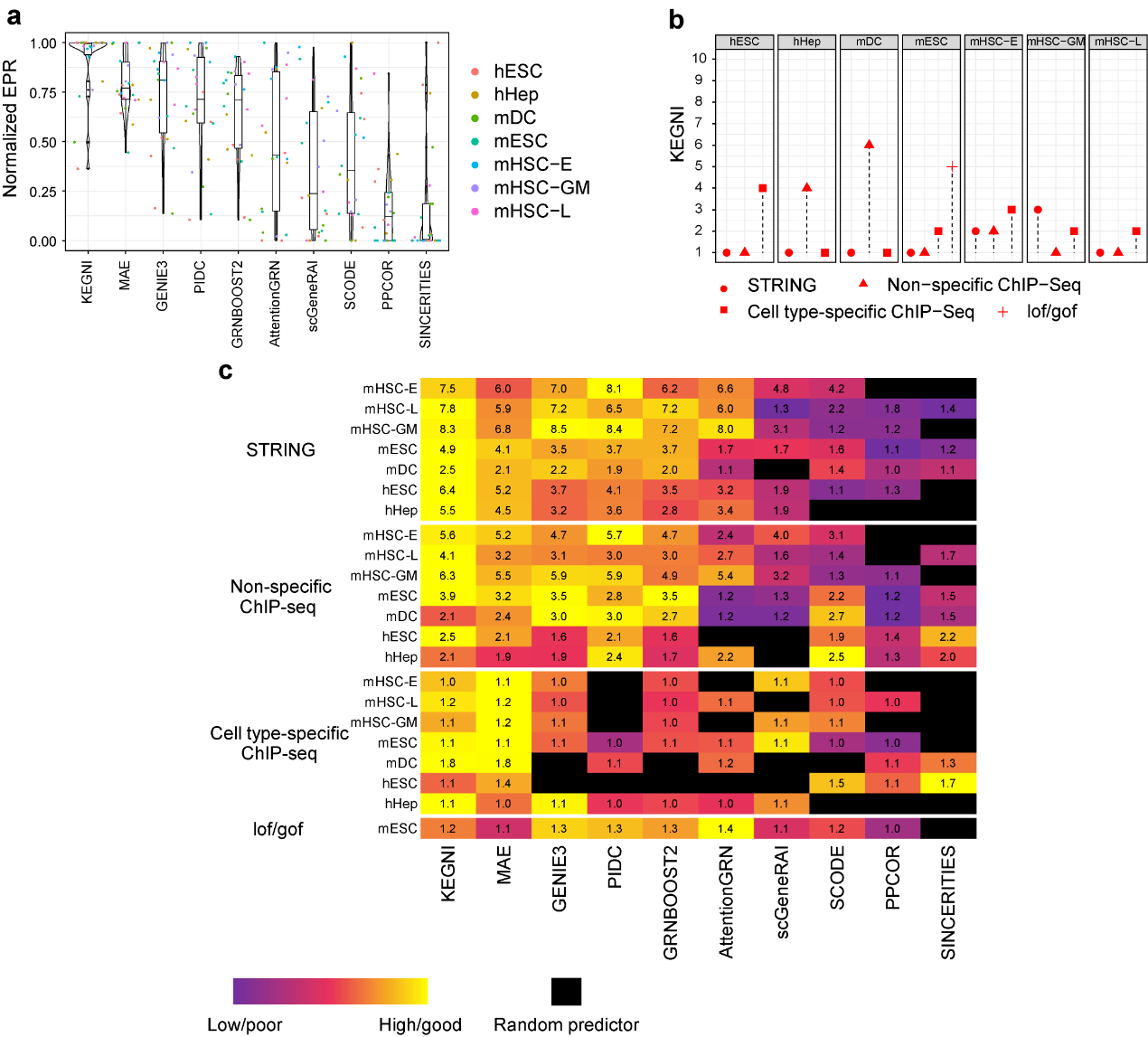


**Fig. S4.** Performance evaluation of KEGNI and other methods in GRN inference on datasets with TFs and the 1000 most variable genes. **a.** Violin plots of normalized EPR values across methods. The plot indicates the median values from ten repetitions. Colors represent distinct datasets, including human embryonic stem cells (hESC), human mature hepatocytes (hHep), mouse dendritic cells (mDC), mouse embryonic stem cells (mESC), and three lineages of mouse hematopoietic stem cells (mHSC), namely erythroid lineage (mHSC-E), granulocyte-macrophage lineage (mHSC-GM) and lymphoid lineage (mHSC-L). **b.** Ranking results of KEGNI compared to other methods across all datasets and their corresponding ground truths. Rankings are visualized using different shapes, reflecting the sources of ground-truth networks: circles represent functional interaction networks from the STRING database, triangles denote non-specific ChIP-seq networks, squares indicate cell type-specific ChIP-seq networks, and plus signs (+) represent loss-of-function/gain-of-function networks. **c.** Detailed EPR results for each dataset. Each row represents a scRNA-seq dataset, with colors scaled between 0 and 1 using the minimum-maximum scaling on EPR values. Values below the performance of a random predictor are indicated by black squares.


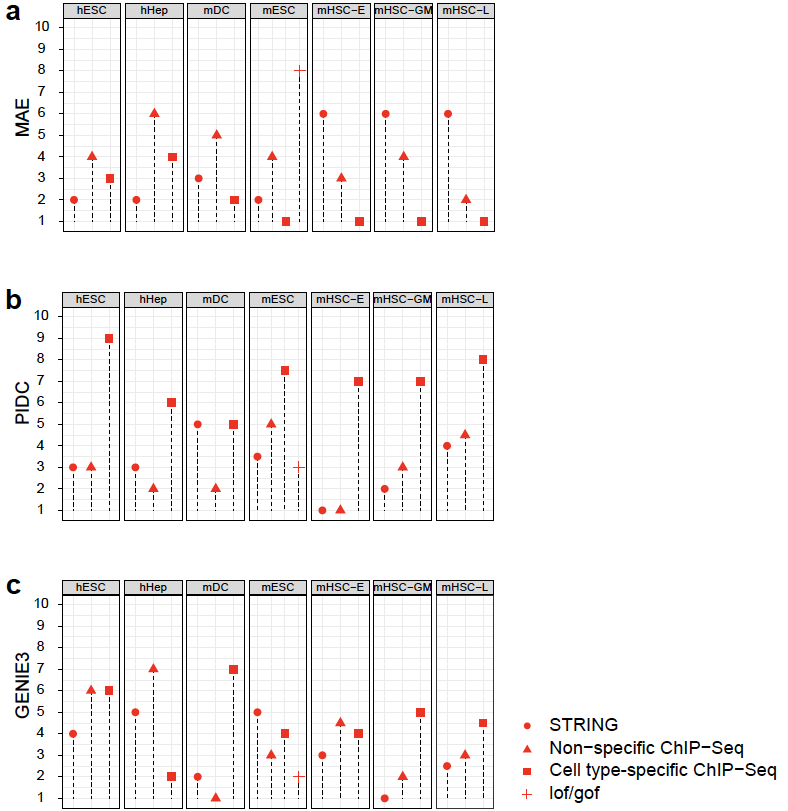


**Fig. S5.** Ranking results of MAE (**a**), PIDC (**b**), and GENIE3 (**c**) across all datasets with TFs and the 1000 most variable genes. Rankings are visualized using different shapes, reflecting the sources of ground-truth networks: circles represent functional interaction networks from the STRING database, triangles denote non-specific ChIP-seq networks, squares indicate cell type-specific ChIP-seq networks, and plus signs (+) represent loss-of-function/gain-of-function networks.


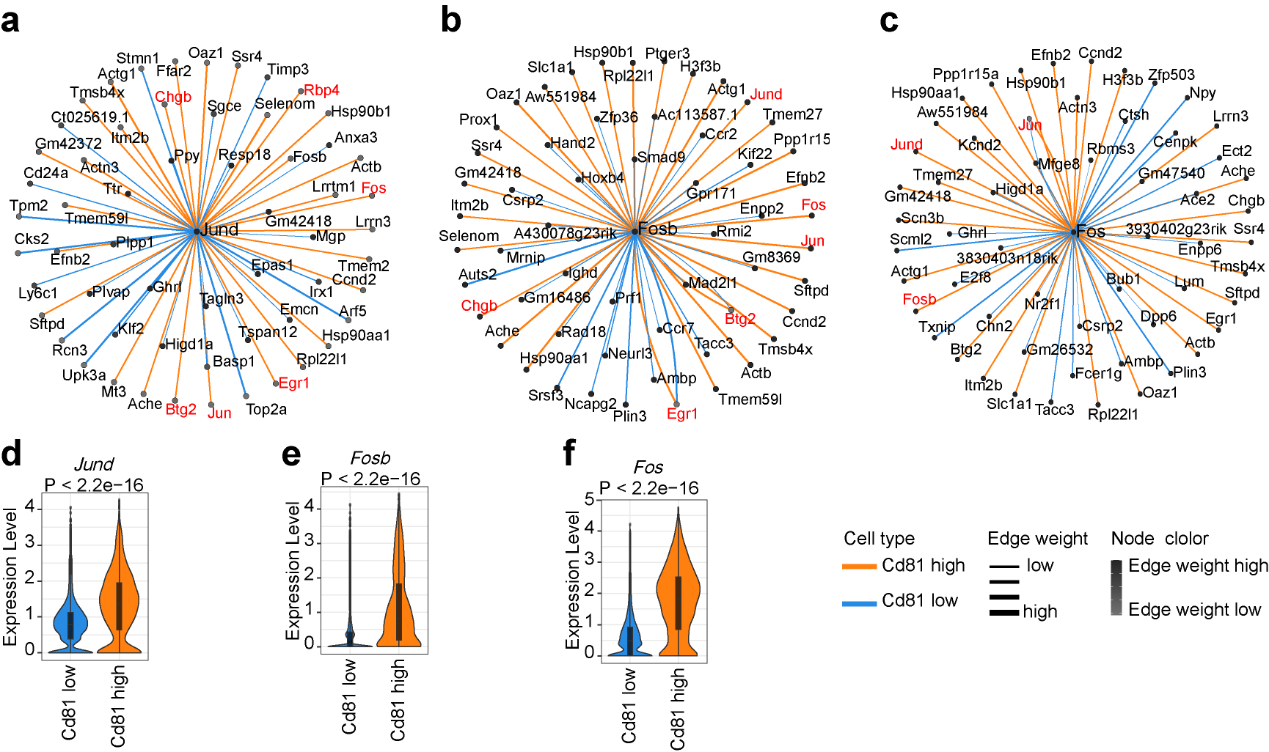


**Fig. S6.** Subnetworks of *Jund* (**a**), *Fosb* (**b**), *Fos* (**c**). Violin plots showing expression levels of *Jund* (**d**), *Fosb* (**e**), and *Fos* (**f**) in *Cd81*^high^ and *Cd81*^low^ subpopulations. P-values were calculated using an unpaired Wilcoxon rank-sum test. Nodes represent genes, and edges are colored yellow for *Cd81*^high^ and blue for *Cd81*^low^, with edge thickness corresponding to edge weight.


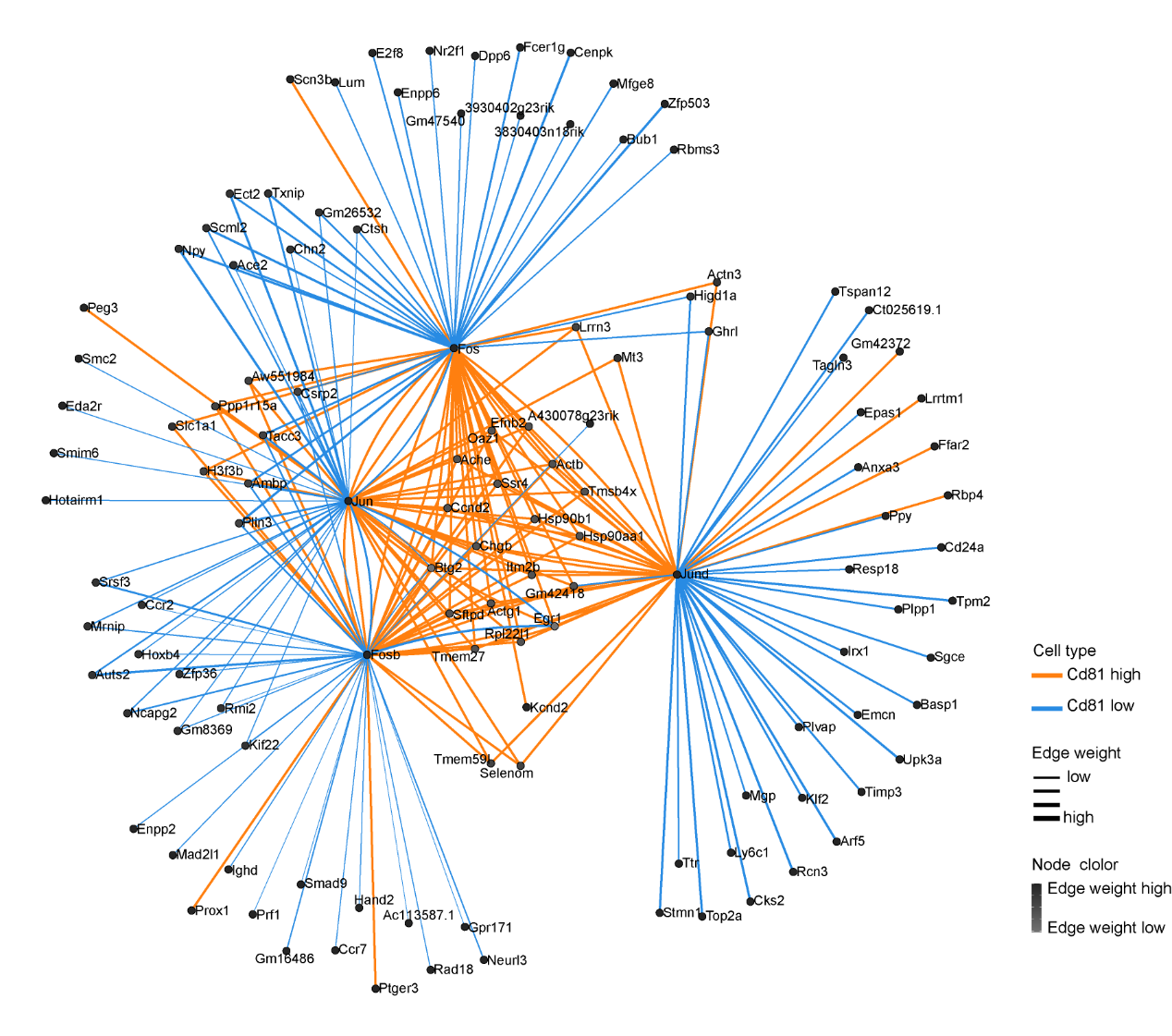


**Fig. S7.** Subnetwork of AP-1 components. The figure depicts the subnetwork of *Jun*, *Jund*, *Fos*, and *Fosb*, which form the heterodimeric transcription factor AP-1.

**Supplementary Notes**

**Supplementary Note 1. Descriptions of different databases that can be used for knowledge graph construction.**

**KEGG pathway database**

KEGG pathway database

KEGG (https://www.kegg.jp) is a database resource for representation and analysis of biological systems, consisting of sixteen manually curated databases for various data objects [32, 33]. Among them, KEGG PATHWAY is a collection of manually drawn pathway maps representing our knowledge of the molecular interaction, reaction and relation networks. The update history of KEGG pathway maps is publicly available at: <https://www.kegg.jp/kegg/docs/upd_map.html>.

The KEGG Markup Language (KGML) is an exchange format of the KEGG graph objects, especially the KEGG pathway maps that are manually drawn and updated. The KGML files contain computerized information about graphical objects and their relations in the KEGG pathways as well as information about orthologous gene assignments in the KEGG GENES database. KGML files can be downloaded programmatically by following the instructions provided in the documentation (<https://www.kegg.jp/kegg/rest/keggapi.html>).

**TRRUST database**

TRRUST is a manually curated database. TRRUST v1 contains 8,015 transcriptional regulatory relationships between 748 human transcription factors (TFs) and 1,975 non-TF genes [30]. TRRUST v2 contains 8,444 and 6,552 TF-target regulatory relationships of 800 human TFs and 828 mouse TFs, respectively [29]. The most recent update of TRRUST was on Nov 23, 2021. The regulatory information curated in TRRUST v2 can be downloaded from: https://www.grnpedia.org/trrust/downloadnetwork.php.

**RegNetwork database**

RegNetwork (http://www.regnetworkweb.org) is a knowledge-based gene regulatory network database for human and mouse, integrating both transcriptional and post-transcriptional regulatory relationships [31]. The database includes three major types of entities: transcription factors (TFs), microRNAs (miRNAs), and target genes. It collects curated regulatory interactions from 25 publicly available databases, covering experimentally validated and computationally predicted interactions. In addition to documented regulations, RegNetwork also incorporates inferred regulatory relationships based on transcription factor binding site (TFBS) motifs. Since RegNetwork was published in 2015 and has not incorporated more recent regulatory information, we did not include this database in our knowledge graph construction, finally.

For both KEGG PATHWAY and TRRUST, the compiled and processed regulatory information used for knowledge graph construction can be downloaded from Zenodo (https://zenodo.org/records/15711227).

**Supplementary Note 2. Construction of the cell type-specific knowledge graph with cell type-specific markers.**

To construct the cell type-specific knowledge graph, it is essential to identify marker genes for each cell type. We downloaded all cell type-specific markers across various human and mouse tissues from CellMarker 2.0 [37] and adopted a three-step filtering strategy to obtain markers for each cell type.

1. We retained only marker genes annotated as “normal” cell types and classified as protein-coding genes. This step removed both cancer-associated markers and non-coding RNAs. This filtering step was applied universally across all datasets.

2. We further filtered the markers for each dataset based on its biological context, including species, tissue class, and tissue type.

3. For datasets with clearly defined cell type annotations, we further matched markers using the specific cell names.

To illustrate this process more concretely, we describe how this pipeline was applied to the PBMC, mDC [78], and hESC [80] datasets used in this study:

1. PBMC dataset: This dataset includes well-defined immune cell types (e.g., naive CD4+ T cell, naïve B cell, myeloid dendritic cell, classical monocyte). Markers were filtered using the following criteria: species = human, tissue class = Blood, tissue type = Peripheral blood, cancer type = Normal, gene type = protein-coding. Furthermore, we selected markers corresponding specifically to the following cell names: naïve CD4+ T cell, classical monocyte, naïve B cell, and myeloid dendritic cell (https://support.10xgenomics.com/single-cell-multiome-atac-gex/datasets).

2. mDC dataset: For this dataset, we selected markers with species = mouse, tissue class and tissue type = bone marrow, cancer type = Normal, and gene type = protein-coding. Since this dataset does not provide finer cell type annotations within mDCs, we retained markers associated with all types of dendritic cells, such as conventional dendritic cells and immature dendritic cells.

3. hESC dataset: This dataset describes the differentiation trajectory of human embryonic stem cells (hESCs) into definitive endoderm through mesendoderm. We selected markers with species = human, tissue class = embryo, and cancer type = Normal, and retained seven relevant tissue types: Embryo, Embryonic stem cell, Posterior presomitic mesoderm, Anterior presomitic mesoderm, Presomitic mesoderm, Endoderm, and Mesoderm. Only protein-coding markers were retained. We further filtered the markers by cell name, keeping those related to embryo, stem cells, mesoderm, and endoderm. All cell type-specific marker gene lists used in this study are publicly available in the Zenodo repository (https://zenodo.org/records/15711227).

After obtaining the final set of marker genes for each cell type, we filtered the base knowledge graph to construct the cell type-specific graph. Specifically, we retained nodes corresponding to the cell type marker genes and subsequently included their first-order neighbors (that is, genes directly connected to the marker genes). All cell type-specific knowledge graphs used in this study are publicly available in our GitHub repository (https://github.com/Lipxiao/KEGNI). To further support reproducibility and facilitate the application of our approach to other datasets, we provide an R Markdown file that demonstrates the complete construction process of a cell type-specific knowledge graph based on a given marker gene list, using the native CD4 T cell dataset as an illustrative example.

**Supplementary Note 3**. **Introduction of knowledge graph embedding methods: TransE and ComplEx.**

A knowledge graph (KG) is a structured representation of entities and their relationships, typically modeled as triples $\left( h,r,t \right)$, where $h$ and $t$ denote head and tail entities, and $r$ represents the relation between them. Knowledge graph embedding (KGE) methods aim to learn low-dimensional vector representations of entities and relations while preserving the graph’s structural and semantic properties.

TransE, introduced by Bordes et al. in 2013 [75], models relationships as translations in the embedding space. Specifically, for a triple $\left( h,r,t \right)$, the embedding of the tail entity $t$ is expected to be close to the embedding of the head entity $h$ plus the embedding of the relation $r$, formulated as $h+r\approx t$. The scoring function measures the plausibility of a triple by the negative distance:

$$\begin{aligned} d_{r}\left( h,t \right)=\gamma-\left| \left| h+r-t \right| \right|\#\left( 1 \right) \end{aligned}$$

where $\gamma$ is a fixed margin hyperparameter.

TransE is appreciated for its simplicity and computational efficiency and performs well on one-to-one relationships. However, its linear translation assumption limits its ability to model complex relational patterns such as one-to-many, many-to-one, and antisymmetric relations [76].

ComplEx, introduced by T. Trouillon et al. in 2016 [74], addresses the limitations of real-valued embedding models by embedding entities and relations into a complex vector space. In ComplEx, entities and relations are embedded as complex-valued vectors, and the scoring function is defined as:

$$\begin{aligned} d_{r}\left( h,t \right)=\text{Re}\left( h^{\top}diag\left( r \right)\bar{t} \right)\#\left( 2 \right) \end{aligned}$$

In this equation, $Re$ denotes the real part of a complex number, and $\overline{t}$ represents the complex conjugate of the tail entity’s embedding $t$. The term $diag\left( r \right)$ represents a diagonal matrix formed from the complex-valued relation embedding vector $r$. Multiplying this diagonal matrix between vectors $h^{\top}$ and $\overline{t}$ is mathematically equivalent to computing the element-wise product among $h,r,\overline{t}$, followed by a summation over dimensions:

$$\begin{aligned} d_{r}\left( h,t \right)=\text{Re}\left( \sum_{i=1}^{d} h_{i}\cdot r_{i}\cdot\overline{t_{i}} \right)=\mathrm{Re}\left( <h,r,\overline{t}> \right)\#\left( 3 \right) \end{aligned}$$

A notable advantage of ComplEx is its capability in handling complex relation types, including one-to-many, many-to-many, symmetric, and antisymmetric relations, using complex-valued embeddings and conjugate operations. However, ComplEx requires higher computational demands compared to real-valued models. Moreover, the operations within the complex space can be less intuitively interpretable than the vector translation mechanisms used in models such as TransE [74, 77].
